# Role of endoplasmic reticulum Ca2+ signaling in the pathogenesis of Alzheimer disease

**DOI:** 10.3389/fnmol.2013.00029

**Published:** 2013-09-18

**Authors:** Elena Popugaeva, Ilya Bezprozvanny

**Affiliations:** ^1^Laboratory of Molecular Neurodegeneration, Saint Petersburg State Polytechnical UniversitySaint Petersburg, Russia; ^2^Department of Physiology, University of Texas Southwestern Medical Center at DallasDallas, TX, USA

**Keywords:** Alzheimer disease, Ca^2^^+^ signaling, presenilins, endoplasmic reticulum, inositol trisphosphate receptors, ryanodine receptors, store-operated Ca^2^^+^ channels, dantrolene

## Abstract

Alzheimer disease (AD) is a major threat of twenty-first century that is responsible for the majority of dementia in the elderly. Development of effective AD-preventing therapies are the top priority tasks for neuroscience research. Amyloid hypothesis of AD is a dominant idea in the field, but so far all amyloid-targeting therapies have failed in clinical trials. In addition to amyloid accumulation, there are consistent reports of abnormal calcium signaling in AD neurons. AD neurons exhibit enhanced intracellular calcium (Ca^2^^+^) liberation from the endoplasmic reticulum (ER) and reduced store-operated Ca^2^^+^ entry (SOC). These changes occur primarily as a result of ER Ca^2^^+^ overload. We argue that normalization of intracellular Ca^2^^+^ homeostasis could be a strategy for development of effective disease-modifying therapies. The current review summarizes recent data about changes in ER Ca^2^^+^ signaling in AD. Ca^2^^+^ channels that are discussed in the current review include: inositol trisphosphate receptors, ryanodine receptors, presenilins as ER Ca^2^^+^ leak channels, and neuronal SOC channels. We discuss how function of these channels is altered in AD and how important are resulting Ca^2^^+^ signaling changes for AD pathogenesis.

## INTRODUCTION

Calcium (Ca^2+^) is one of the most important second messengers in the nervous system. Ca^2+^-mediated signal transduction connects membrane excitability and biological functions of neurons ranging from proliferation, secretion, gene expression, ATP production, cell death to memory formation and its loss. Acting at the border of electrical and signaling “worlds” of the cell, Ca^2+^-permeable channels play a major role in many key aspects of neuronal functions. Due to the huge importance of the calcium as the second messenger neurons utilize many approaches to regulate intracellular Ca^2+^ content, mainly via local signal transduction pathways. Neuronal Ca^2+^ influx can be maintained by different Ca^2+^-permeable channels, such as voltage-gated Ca^2+^ channels of plasma membrane, *N*-methyl-D-aspartate (NMDA) receptors, α-amino-3-hydroxy-5-methyl-4-isoxazolepropanoic acid (AMPA) receptors, nicotinic receptors, store-operated Ca^2+^ channels (SOC). Ca^2+^ can also be released from intracellular stores of endoplasmic reticulum (ER) via inositol-1,4,5-trisphosphate receptors (InsP3R) and ryanodine receptors (RyanRs). Mitochondria also play an important role in intracellular Ca^2+^ handling. Neurons are highly susceptible to any changes in intracellular Ca^2+^ concentrations: insufficient intracellular Ca^2+^ content lead to abnormal functioning of neurons, whereas excessive Ca^2+^ levels cause cell death (Berridge, 1998). Therefore, even small fluctuations in Ca^2+^ content can be very detrimental over long life of a neuron (Khachaturian, 1989).

Alzheimer disease (AD) is the threat of twenty-first century that is responsible for the majority of senile dementia. AD progresses slowly and affects neurons in the brain. Currently there are two main proteins whose dysfunctions and accumulation in the brain are correlated with the disease progress. The first is 40–42 long beta-amyloid (Aβ) peptides that constitute a major part of neuritic plaques and cause excessive neurotoxicity. These peptides are cleaved from the amyloid precursor protein (APP) by β- and γ-secretases (Hardy and Selkoe, 2002). The second protein is tau whose hyperphosphorylation results in misfolding and forming of proteolysis-resistant neurofibrillar tangles (NFTs). Aβ40, Aβ42, and NFT are synaptotoxic to neurons and facilitate cell death (Small and Duff, 2008). Although the exact mechanism how Aβ40, Aβ42, and NFT mediate AD pathogenesis is not fully understood, there are observations that link Aβ42 accumulation with elevated Ca^2+^ levels in neuronal cytoplasm *in vivo* (Kuchibhotla et al., 2008). It has been shown that oligomers of Aβ is able to make Ca^2+^ permeable channels in plasma membrane of neurons, therefore directly affecting intracellular Ca^2+^ concentration (Arispe et al., 1993). Recent publications state that soluble oligomeric form of Aβ42 potentiate Ca^2+^ liberation from the ER through the stimulated production of inositol trisphosphate (Demuro and Parker, 2013) and by stimulating synaptic mGluR5 receptors (Renner et al., 2010).

There is another line of evidence coming from mouse models harboring presenilin’s mutations that AD-like symptoms and synaptic dysfunction can occur due to Ca^2+^ accumulation in the ER in the absence of Aβ pathology (Stutzmann et al., 2004; Chakroborty et al., 2009; Zhang et al., 2010b). Early changes in intraneuronal Ca^2+^ regulation are common observations in AD patients (Emilsson et al., 2006; Stutzmann, 2007; Bezprozvanny and Mattson, 2008). All these observations support calcium hypothesis of AD. This hypothesis was first formulated in 1987 by Dr. Zaven Khachaturian who proposed that sustained changes in intracellular calcium homeostasis provide the final common pathway for AD and age-associated brain changes (Khachaturian, 1987). Since that time many advances in our understanding of Ca^2+^ signaling in AD have been obtained. New Ca^2+^ permeable channels have been identified, some of them directly linked to AD. For example, it has been demonstrated that presenilins encode passive ER Ca^2+^ leak channels (Tu et al., 2006) and a novel Ca^2+^ channel called Ca^2+^ homeostasis modulator 1 (CALHM1) has been linked to late-onset AD by genetic evidence (Dreses-Werringloer et al., 2008). However, as it usually happens with new findings, the existence of these novel Ca^2+^ channels and their role in AD has been challenged. The main purpose of the current paper is to review recent publications in the field of ER Ca^2+^ signaling in the context of AD pathology. We will review the role of two well accepted ER Ca^2+^ channels that release Ca^2+^ out of the neuronal ER – InsP_3_R and RyanR. We will also discuss new findings about the role of presenilins and neuronal SOC in neuronal function. Our focus will be on potential role of these channels in AD pathology and as targets for development of disease-modifying therapies.

## INOSITOL TRISPHOSPHATE RECEPTORS

The first observation of exaggerated InsP3R-mediated Ca^2+^ release from ER in fibroblasts from AD patients has been obtained even before the identification of presenilins (Ito et al., 1994). It was later shown that these fibroblasts (from patients AG06840 and AG06848) harbor A246Q mutation in PSEN1 (description in Coriell Institute Cell Database). The studies with fibroblasts taken from PS1-M146V knockin mice and with *Xenopus* oocytes expressing human presenilin proteins 1 and 2 (PS1 and PS2) mutant constructs showed an upregulation of InsP3R-mediated Ca^2+^ release (Leissring et al., 1999a, b, 2000). Experiments in cortical neurons using whole-cell patch clamp and rapid Ca^2+^ imaging in brain slices from mutant PS1-M146V mice also demonstrated almost threefold exaggeration of ER Ca^2+^ liberation by photolysis of caged InsP3 and accompanying enhancement of Ca^2+^-evoked outward membrane currents (Stutzmann et al., 2004). Similar results of enhanced InsP3-evoked Ca^2+^ signals were observed in 3xTg-AD mice (Stutzmann et al., 2006). Important to note that the Ca^2+^ disturbances were already observed in the 3xTg-AD mice at the age of 4–6 weeks that precedes appearance of Aβ plaques and NFTs by several months (Oddo et al., 2003). Later on it has been reported that in non-neuronal DT40 and Sf9 cell models familial AD (FAD) associated mutations PS1-M146L and PS2-N141I interact with InsP3R and exert stimulatory effects on its gating activities (Cheung et al., 2008). In more recent study the same group has proposed that stimulation of InsP3R gating by expression of mutant PS1-M146L in DT40 and PC12 cells results in generation of reactive oxygen species (ROS; Muller et al., 2011). Authors report that exaggerated Ca^2+^ signaling through InsP3R-PS interaction and generation of ROS may contribute to the pathology of AD (Muller et al., 2011).

**FIGURE 1:**
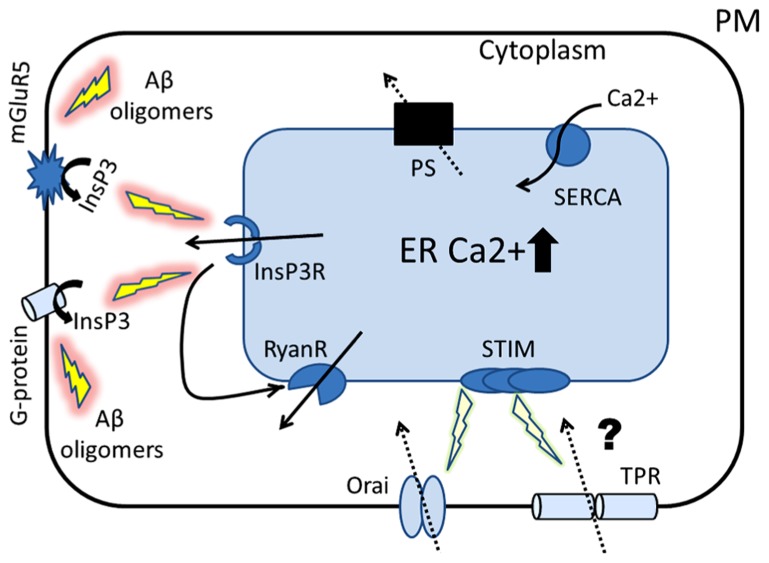
**Proposed role of ER Ca^2+^ signaling in the pathogenesis of AD.** The cartoon represents the Ca^2+^ hypothesis of AD that places presenilins (PS) in the center of AD pathogenesis. Amyloid oligomers stimulate InsP3R-mediated Ca^2+^ release from ER by activating synaptic mGluR5 receptors and by stimulating G-protein-mediated InsP3 production. Our laboratory has shown that presenilins function as ER Ca^2+^ leak channels and FAD associated PS mutations disrupt this function, causing overloading of ER with Ca^2+^. Similar ER Ca^2+^ overload occurs as a result of neuronal aging process. The first physiological response to ER Ca^2+^ elevation is compensatory increase in expression and/or activity of inositol trisphosphate receptors (InsP3R) and ryanodine receptors (RyanRs). The second response to ER Ca^2+^ overload is reduction in store-operated Ca^2+^ (SOC) entry, a mechanism involved in refilling of ER Ca^2+^ stores (mediated by Orai and TRP channels). We hypothesize that these initially compensatory and protective mechanisms of ER Ca^2+^ signaling become pathogenic in aging neurons and eventually lead to synaptic dysfunction, synaptic loss and neurodegeneration.

Important to note the recent study showing that intracellular application of Aβ oligomers into *Xenopus* oocytes stimulates G-protein-mediated InsP3 production and consequent Ca^2+^ release from the ER, that is cytotoxic (Demuro and Parker, 2013; depicted in **Figure 1**). Also, it was reported that Aβ oligomers stimulate synaptic mGluR5 receptors linked with InsP3 production (Renner et al., 2010). Although detrimental effect of Aβ oligomers on neurons has been extensively studied and many publications demonstrated that Aβ aggregates promote the increase in cytosolic Ca^2+^ content of neurons (Walsh et al., 2002; Demuro et al., 2005, 2010; Deshpande et al., 2006; Simakova and Arispe, 2007; Bezprozvanny and Mattson, 2008; Green and LaFerla, 2008; Kuchibhotla et al., 2008), the exact mechanism how Aβ contributes to disruption of Ca^2+^ signaling is not known. Therefore, the studies of Demuro and Parker (2013) and Renner et al. (2010) could potentially provide a connection between amyloid and overactivation of InsP3R-mediated Ca^2+^ signals.

## RYANODINE RECEPTORS AND EFFECTS OF DANTROLENE

Ryanodine receptors are expressed in soma, proximal dendrites as well as in distal processes and spines. RyanRs activity is enhanced in dendrites and synaptic spines from presymptomatic 3xTg-AD and TASTPM (APPsw; PS1.M146V; Howlett et al., 2004) AD mice (Goussakov et al., 2010). RyanR-mediated Ca^2+^-induced Ca^2+^ release (CICR) in 3xTg-AD mice is exaggerated in response to synaptic stimulation, including NMDAR-mediated Ca^2+^ influx (Goussakov et al., 2010). These authors proposed that enhanced synaptic CICR may alter synaptic function and may be recognized as an early pathogenic factor in AD (Goussakov et al., 2010). Increased levels of RyanR are at least partially responsible for enhanced CICR in AD neurons. Increased expression of RyanR has been described in human AD cases and in patients with mild cognitive impairment (MCI; Kelliher et al., 1999; Bruno et al., 2012). Elevated RyanR2 expression, cognitive decline, and synaptic loss observed in MCI patients are mirrored by an increase in RyanR2 expression and Ca^2+^ release in presymptomatic AD mice (Kelliher et al., 1999; Stutzmann et al., 2006; Chakroborty et al., 2009; Zhang et al., 2010b). Recently, it has been suggested that increased RyanR expression at early stages of AD might play a role as a compensatory mechanism to stabilize the preexisting synaptic deficits and normalize the depressed synaptic network (Chakroborty et al., 2012b). Similar idea of elevated RyanR3 expression as a neuroprotective response to Aβ1–42 toxic effects has been suggested before (Supnet et al., 2010).

Several studies addressed the role of RyanR in the context of AD by using pharmacological agent dantrolene. Dantrolene is an antagonist of the RyanR and is used clinically to treat malignant hyperthermia, neuroleptic malignant syndrome, and muscle spasms (Krause et al., 2004; Inan and Wei, 2010). In the first study the dantrolene was administered to 3xTg-AD mice by intracerebroventricular (ICV) injection for 3 months using an Alzet intracranial ventricular infusion system and then subcutaneously three times per week for 8 month (Peng et al., 2012). The authors state that dantrolene treatment significantly reduced both memory deficits tested by Morris water maze test and amyloid plaque load in the hippocampus in 13-month-old 3xTg-AD mice (Peng et al., 2012). The second work performed sub-chronically short-term (4 weeks) treatment of AD models (3xTg-AD and TASTPM) with dantrolene (Chakroborty et al., 2012a). Using two-photon Ca^2+^ imaging and patch clamp recordings authors showed that dantrolene treatment normalized ER Ca^2+^ signaling within somatic and dendritic compartment in early and late-stage AD mice in hippocampal slice experiments (Chakroborty et al., 2012a). The third study (Oules et al., 2012) was performed with transgenic mice expressing human APPswe mutation (Tg2576). These authors observed that dantrolene treatment diminished Aβ load, reduced histological lesions, and slowed down learning and memory deficits in Tg2576 mice (Oules et al., 2012). These studies suggested that inhibition of RyanR with dantrolene may exert beneficial effects in the context of AD pathology. However, opposite conclusion was obtained by our laboratory in experiments with APPPS1 transgenic mouse model (Thy1-APPKM670/671NL, Thy1-PS1L166P; Zhang et al., 2010b). In these studies we discovered that long-term (starting at 2 months of age) oral feeding of dantrolene exacerbated plaque formation and resulted in loss of hippocampal synaptic markers and neuronal deterioration in 8-month-old APPPS1 mice (Zhang et al., 2010b). How can these seemingly divergent observations that center on dantrolene be explained? It is difficult to directly compare these results due to different routes of dantrolene administration used in the studies, variability in duration of treatments, mice age groups, and different AD mouse models used in the studies. Another potential problem with interpreting these results is that specific RyanR inhibitors do not exist and the drug dantrolene used in most studies has additional targets such as store-operated Ca^2+^ channels (Zhao et al., 2006). Moreover, dantrolene is specific for skeletal muscle RyanR1 (Krause et al., 2004), and does not block neuronal RyanR2 and RyanR3 subtypes effectively. To resolve this controversy, our laboratory is currently taking a genetic approach to evaluate a role of RyanRs in AD. Our initial results indicate that RyanR may play initially compensatory and later detrimental role in the context of AD pathology.

Taking together, it is clear from multiple studies with various AD cellular and animal models that ER Ca^2+^ signaling is disturbed in AD and that activity of both InsP3R and RyanR is enhanced. Increased expression of RyanRs at least partially responsible for enhanced CICR in AD neurons. The mechanisms responsible for enhanced activity of InsP3R are less certain and may involve direct gating of InsP3R by presenilins. It is also likely that increased ER Ca^2+^ levels contribute to enhanced RyanR-mediated and InsP3R-mediated Ca^2+^ release, as discussed in more details in the following section. It also appears that RyanR is a potential pharmacological target for AD treatment and that dantrolene may provide potential avenue for suppressing RyanR activity in AD.

## PRESENILINS

There are mutations in presenilin 1 (*PSEN1*), presenilin 2 (*PSEN2*), and *APP* genes that are linked to early onset FAD. The majority, nearly 200, of these mutations are within *PSEN1*. To date many known *PSEN1* mutations contribute to Ca^2+^ disruptions in ER Ca^2+^ signaling (Bezprozvanny and Mattson, 2008). PS1 and PS2 constitute the catalytic pore of the γ-secretase complex, other partner of the complex are nicastrin, aph-1, and pen-2 (De Strooper, 2003). The γ-secretase complex cleaves type-1 transmembrane proteins, including Notch receptor protein and APP. One of the main therapeutic approaches to AD is focused on development of γ-secretase inhibitors (GSIs) and modulators, however so far this approach has failed in phase III clinical trials of Eli–Lilly’s Semagacestat, a non-selective GSI (Doody et al., 2013). Semagacestat treatment resulted in worsen cognition scores and increase in the risk of skin cancer (Doody et al., 2013), most likely due to inhibition of Notch processing. As a result, clinical trials of GSIs have been halted.

In addition to contributing to altered γ-secretase function in AD pathogenesis, FAD PS mutations result in disturbed Ca^2+^ signaling in neurons (reviewed in Stutzmann, 2007; Bezprozvanny and Mattson, 2008; Supnet and Bezprozvanny, 2010a, b). As discussed above, multiple studies demonstrated enhanced InsP3R-mediated and RyanR-mediated ER Ca^2+^ release in PS-FAD cells. Presenilin mutations also affected SOC, a refilling mechanism for ER stores (Leissring et al., 2000; Yoo et al., 2000; Giacomello et al., 2005; Zhang et al., 2010b). To explain these findings, it was suggested that gating of InsP3R or RyanRs directly modulated by presenilins (Cheung et al., 2008, 2010; Rybalchenko et al., 2008). It was also suggested that presenilins potentiate activity of sarco-/endoplasmic reticulum Ca^2+^ ATPase (SERCA; Green et al., 2008), a mechanism that could contribute to the overfilling of ER Ca^2+^ store.

Our laboratory offered an alternative mechanistic explanation for most of these findings by demonstrating that wild type PSs function as ER Ca^2+^ leak channels (Tu et al., 2006), which function to maintain ER Ca^2+^ homeostasis by constantly leaking Ca^2+^ into the cytosol and balancing SERCA activity. Our results suggested that presenilin holoproteins function as low conductance passive ER Ca^2+^ leak channel, and that ER Ca^2+^ leak function of presenilins does not depend on their γ-secretase activity (Tu et al., 2006). Moreover, we found that some, but not all, FAD PS mutations disrupt Ca^2+^ leak function (Tu et al., 2006; Nelson et al., 2007, 2010), leading to the overfilling of ER with Ca^2+^ and exaggerated ER Ca^2+^ release observed in PS1/PS2 FAD mutants fibroblasts (Tu et al., 2006; Nelson et al., 2007, 2010), cultured hippocampal neurons from 3xTg AD neurons (Zhang et al., 2010b), and primary lymphoblasts from FAD patients (Nelson et al., 2010). These data suggest that mutations in presenilins directly linked to deranged Ca^2+^ signaling and neuronal dysfunction in AD by causing ER Ca^2+^ overload. Our hypothesis has been directly challenged, in particular by the group of Dr Kevin Foskett (Shilling et al., 2012). These authors claimed that presenilin does not have a pore and cannot act as an ion channel (Cheung et al., 2008; Shilling et al., 2012). As we previously outlined, a number of serious technical and experimental issues exists with their negative arguments (Bezprozvanny et al., 2012). Other experiments that oppose to our hypothesis have also been reported (Zatti et al., 2004, 2006). In contrast to our finding, the authors of these papers observed that FAD-PS expression lower the ER calcium content (Zatti et al., 2004, 2006). Despite existence of these controversial results independent experimental support for leak function of presenilin recently began to accumulate (Das et al., 2012). In a recent study, Bandara et al. (2013) performed an unbiased RNAi-based screen for modulators of calcium homeostasis in HEK293 cells. They transfected 250 candidate short-interfering RNAs (siRNAs) into the cells and used the mathematical model to quantify the effects of knockdown on calcium pump and leak rates, which resulted in the identification of proteins involved in the elusive ER Ca^2+^ leak pathway. Knocking down presenilin-2 or ORAI2 dramatically reduced ER calcium leak rate, and knocking down PEN-2, encoded by *PSENEN*, greatly increased calcium leak rate (Bandara et al., 2013). Knockdown of PSENEN would inhibit proteolytic processing of presenilins and thus increase the holoprotein form of the protein, which is the form of presenilins that functions in ER calcium leak according to our previous experiments (Tu et al., 2006). Thus, enhanced ER calcium leak resulting from PEN-2 knockdown most likely reflects the accumulation of the presenilin holoprotein in the ER. As discussed in the recent review article (Bezprozvanny, 2013) these findings provide strong support to our hypothesis that presenilin holoprotein functions as ER calcium leak channel. Interestingly, Honarnejad et al. (2013) recently reported that there is PS holoprotein upregulation in human AD brain samples, suggesting a possibility of compensatory upregulation of leak pathway in AD neurons in order to reduce ER Ca^2+^ overload.

Where is an ion conduction pore of presenilin leak channel? From the structural-functional analyses we suggested that transmembrane domains 7 and 9 but not transmembrane domain 6 may play a role in forming the ion conductance pore of PS1 (Nelson et al., 2011). Recent publication reported the first crystal structure of archeal homolog of presenilin (PSH; Li et al., 2013). These authors discovered that PSH has a large hole that transverse the entire protein and is surrounded by transmembrane domains 2, 3, 5, and 7. These data are in good agreement with our mutagenesis mapping studies (Nelson et al., 2011). Moreover, these authors postulate that the hole is large enough to allow passage of the small ions (Li et al., 2013), suggesting that PSH may function as an ion channel. Importantly, the motifs that constitute catalytic core are conserved between PSH and PS1, therefore the structure of PS1 should be very similar to the structure of PSH.

## STORE-OPERATED CALCIUM CHANNELS

Recent growing evidence suggests that SOC channels may be involved in AD pathogenesis. SOC channels are unique in the nature of their activation. They are activated in response to lowering of Ca^2+^ content in ER. The first reports about role of SOC channels in the pathogenesis of AD have been published in 2000. Leissring et al. (2000) observed that fibroblasts isolated from PS1-M146V knock in mice exhibit significant impairments in store-operated Ca^2+^ entry after stimulation of cells with bradykinin. These authors suggested that impaired SOC in these cells is due to elevated ER Ca^2+^ levels in PS1-M146V fibroblasts (Leissring et al., 2000). In the same year Yoo et al. (2000) reported alteration in SOC activity in presenilin FAD mutant neurons. Two different mechanisms of mutant PS1-mediated dysregulation of SOC have been proposed (Herms et al., 2003). The first mechanism is linked to direct attenuation of SOC at the cell surface, the second mechanism evokes changes in processing of APP and generation of amyloid peptides (Herms et al., 2003). However, second mechanism cannot explain alterations of SOC observed in the absence of human APP and Aβ42 accumulation. TRP channels may play a role in disruption of neuronal SOC in AD (Yamamoto et al., 2007), but the mechanisms involved in changes in TRP channel expression or activity in AD are poorly understood.

In addition to TRP channels, important players of SOC in excitable and non-excitable cells are stromal interaction molecule 1 and 2 (STIM2) proteins. STIM 1 and STIM2 protein reside in ER, and reduction in ER Ca^2+^ levels causes oligomerization of STIMs, translocation to plasma membrane, and activation of SOC channels (Liou et al., 2005). The molecular identity of neuronal SOC is poorly understood, but most likely involves complex of STIMs with TRP channels and/or Orai proteins (**Figure 1**). Interestingly, changes in expression of STIM1 and STIM2 proteins were found in PS knockout and FAD mutant cells (Bojarski et al., 2009), suggesting a possible mechanism for SOC dysregulation. In recent review articles we suggested a possible connection between dysregulated neuronal SOC and synaptic spine maintenance in AD and aging brains (Popugaeva et al., 2012; Bezprozvanny and Hiesinger, 2013). These ideas are currently being tested experimentally in our laboratory. Another possibility involves potential connection between impaired neuronal SOC and abnormal synaptic vesicle recycling in PS mutant neurons (Zhang et al., 2009, 2010a).

## SUMMARY

In the summary we would like to conclude with our working hypothesis for ER Ca^2+^ dysregulation in AD (**Figure 1**). FAD linked mutations in PS cause disruption of PS Ca^2+^ leak function. As a result Ca^2+^ is accumulating inside of the ER. Similar increase in ER Ca^2+^ levels occur as a result of brain aging. In order to compensate for ER Ca^2+^ overload neurons mount two physiological responses: (1) upregulate gating of InsP3R and expression/activity of RyanR, and (2) downregulate activity of neuronal SOC (**Figure 1**). We hypothesize that these initially protective responses with time become toxic and eventually lead to synaptic dysfunction, synaptic loss, impaired plasticity, and learning, loss of memories and neurodegeneration. The role of RyanR in these processes is likely to be more significant than the role of InsP3R, as InsP3R predominantly localized in the soma, whereas RyanR are abundant in the postsynaptic and presynaptic terminals. Dantrolene provides a possible way to suppress RyanR-mediated Ca^2+^ release pharmacologically, but there are significant issues with specificity of dantrolene effects and its delivery to the brain. Neuronal SOC pathway provides a novel potential target for AD treatment that should be explored further.

## Conflict of Interest Statement

The authors declare that the research was conducted in the absence of any commercial or financial relationships that could be construed as a potential conflict of interest.
